# Recent advances in synthesis and application of perovskite quantum dot based composites for photonics, electronics and sensors

**DOI:** 10.1080/14686996.2020.1752115

**Published:** 2020-05-12

**Authors:** Yaxin Wang, Guanglong Ding, Jing-Yu Mao, Ye Zhou, Su-Ting Han

**Affiliations:** a Institute for Advanced Study, Shenzhen University, Shenzhen, P. R. China; b Shenzhen Key Laboratory of Flexible Memory Materials and Devices, Institute of Microscale Optoelectronics (IMO), Shenzhen University, Shenzhen, P. R. China

**Keywords:** Perovskite quantum dot based composites, thermal stability, chemical stability, photo stability, optoelectronic property, 103 Composites

## Abstract

In recent years, halide perovskite quantum dots (HP-QDs) based composites have been widely developed and used in various applications owing to their unique photonic, electronic and mechanical properties, as well as high stability to water, oxygen, heat and illumination. Remarkable efforts have been made in the synthesis and applications of these materials in photonics, electronics, sensors and other fields. Besides these topics, we also cover enhancement of optoelectronic properties as well as chemical, thermal and photostability of HP-QDs-based composites. We hope this review will promote both the development and applications of perovskite-based materials.

## GRAPHICAL ABSTRACT

**Figure:**
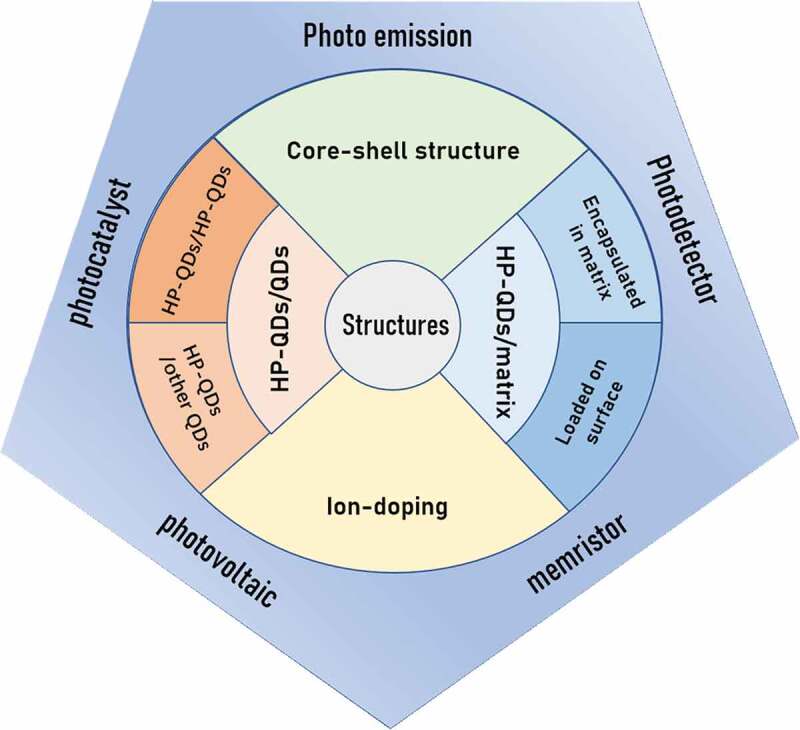

## 1. Introduction

With the rapid development of material science, more and more low-dimension materials, such as nanosheets, nanowires and nanodots, are being developed and applied in various fields due to their unique optical, electrical, mechanical, thermal and other properties [1–11]. Quantum dots (QDs), which are a zero-dimensional (0D) material, have attracted increasing attention as next-generation functional materials. They are widely used for optoelectronic devices including light-emitting diodes [12–14], lasers [15], solar cells [16–18], photodetectors [19] and many other applications including drug delivery, chemical analysis, sensor, bioimaging [20–23]. Due to their quantum confinement effect that appears when the semiconductor materials’ size is smaller than twice its Bohr radius, QDs process excitonic features and enhanced photoluminescence (PL) properties comparing to bulk materials [24–26]. Various types of QDs such as CdS [27], CdSe [13,15,28], ZnS [29], PbS [30], PbSe [31], SnS [32,33], SnSe [34] silicon [35] and halide perovskites have been explored and applied in various applications.

Halide perovskite quantum dots (HP-QDs), with chemical formula of ABX_3_ (A = CH_3_NH_3_, CH_5_N_2,_ Cs; B = Pb, Sn; C = I, Br, Cl), have emerged rapidly as a new outstanding class of QDs materials for their narrower full width at half maximum (FWHW), defect-tolerant structure and high synthesis feasibility comparing to those traditional classes of QDs [36–38]. Owing to their merits, including tunable bandgap, high light-absorption efficiency, low carrier recombination rate, high defect tolerance and high PL quantum yield, HP-QDs have been applied in various electronic and optoelectronic applications such as photoemission [39–41], photovoltaic [42], photodetectors [43], photocatalysts [44] and memristors [45]. For example, Dai et al. had reported HP-QDs exhibiting maximum PL quantum yield up to 100%, showing promising potential for photo-emission [46]. Moreover, the easy and low-cost synthesis earned HP-QDs additional attractions. Via some simple methods such as one-step hot-injection and an even easier ligand-assisted recrystallization at room temperature, ideal HP-QDs with uniform morphology, tunable emission and other superior performance can be obtained [47].

While exhibiting excellent optical properties, the as-obtained HP-QDs suffer from poor chemical, thermal and photostability. Their structure degrades in an atmosphere containing oxygen and water due to photooxidation [48–50]. With an ionic structure and highly dynamic ligand bonding, the photoexcited HP-QDs release electrons that would easily interact with oxygen molecules. The free radicals generated would then react with the amine salt, leading to the decomposition [51]. HP-QDs are also extreme sensitive to many other environmental factors like high temperature and UV light [48,52]. Therefore, improving the environmental stability is always one of the hot topics in the studies of HP-QDs.

**Scheme 1.**
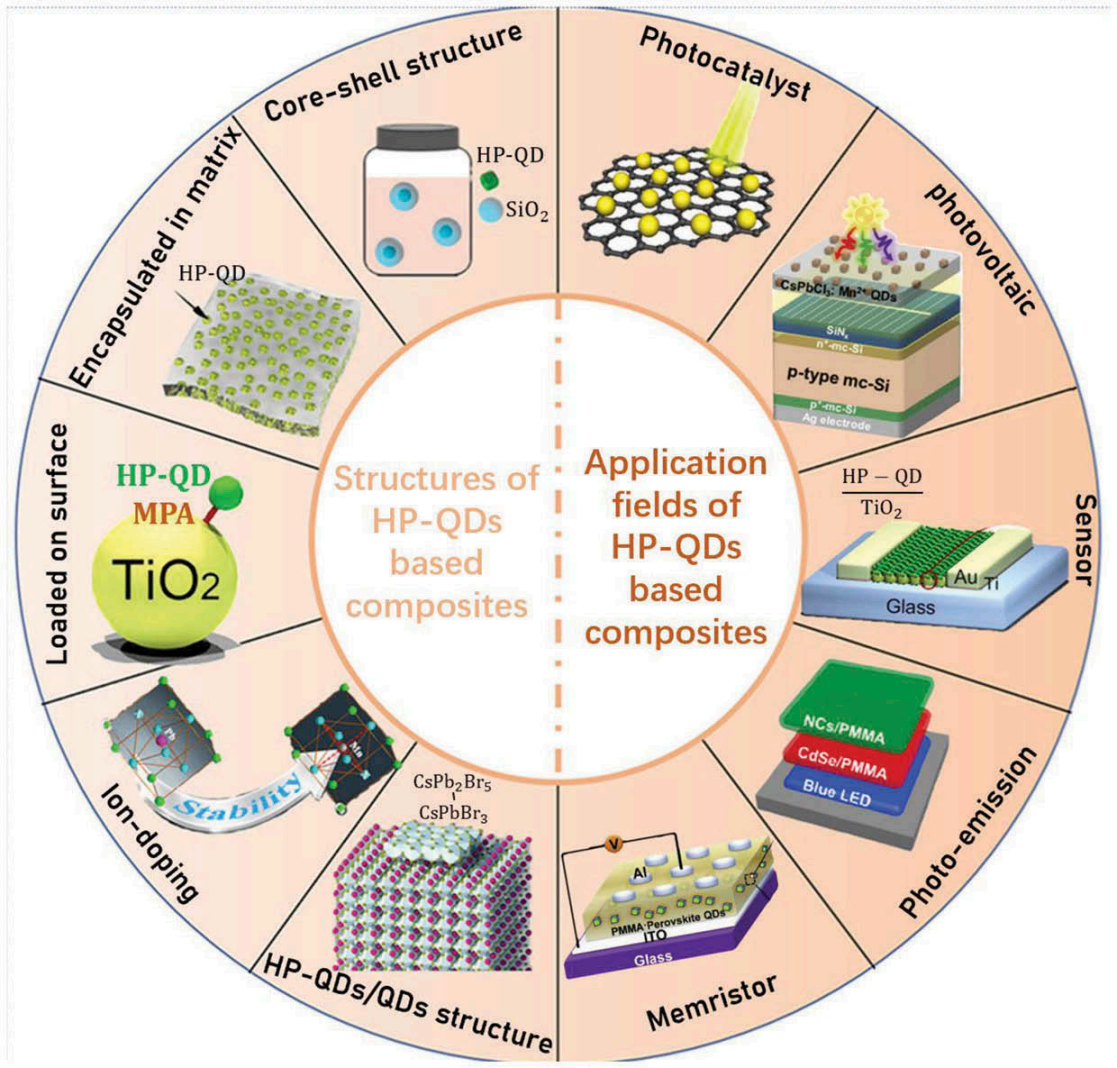
Schematic illustration of the structures and applications of HP-QDs composites discussed in this review. Produced with permission from [69,77]. © 2018 ACS Publications [105,117];. © 2017 Royal Society of Chemistry [116,121]; © 2017 ACS publications [134]; © 2018 ScienceDirect [122,177]; © 2018 ScienceDirect.

To enhance the stability of HP-QDs, various strategies such as shell design, ligand design and overcoating [48] have been explored, of which compositing HP-QDs with other materials to passivate its surface and form a protection layer or heterojunction is a practical and promising one. Various materials including oxides, polymers, metallic ions, and many other organic and inorganic options have been explored in recent years. By diverse structures such as shelling the QDs at single-particle level, encapsulating the QDs into vast matrix, loading QDs onto the surface, ion-doping in the lattice of HP-QDs or forming HP-QDs/QDs nanocomposites, excellent results for HP-QDs based composites have been reported, showing outstanding improvement in stability and some other expected properties for enhanced performance in photonics, electronics, sensors and other fields (as shown in Scheme 1) [53,54]. The two fields of material synthesis methods and material applications can promote the developments of each other. Therefore, it is of great necessity to make a periodic summary in this rapid development of scientific research era. Herein, we systematically discussed the recent development of common fabrication strategies and performances of HP-QDs based composites, and their application in photonics, electronics and sensors.

## 2. Fabrication strategies

### 2.1. Fabrication strategies of halide perovskite quantum dots

The fabrication strategies of HP-QDs can be summarized into two kinds: 1) high-temperature hot-injection (HI) method, which needs serious reaction environment including certain high temperature and protective gas, 2) room temperature (RT) synthesis method, a relatively easy-to-operate and low-cost method [47].

A typical HI synthesis method involves the preparation of required precursor by heating the mixture to a certain temperature under gas protection, and a quick injection into another solution. Via the mechanism of a quick ionic metathesis reaction, the desirable HP-QDs with excellent monodispersity and optical properties can be obtained [55]. Using this method, the nucleation stage happened right after the injection to form nuclei and the growth stage started after its termination, where this separation between two stages allows achieving a narrow size distribution of the nanoparticles [56]. The high reaction temperature also enables better control over the QDs shape and better phase purity [57,58]. CsPbBr_3_ QDs and some other HP-QDs can be obtained using this method [55]. The adjustment of the operating temperature in this process plays an important role of helping control the size of the obtained QDs and would also influence the PL peak position [59,60].

Being less complex, RT synthesis method employs ‘good’ solvent to form the precursor without heating. Then, long-chain organic ligands and ‘poor’ solvent like toluene are mixed with the former solution under intense stirring to facilitate the formation of HP-QDs through recrystallization [61–64]. In this process, the nucleation and growth were started by the instantaneous supersaturation without a separation in timeline as in HI synthesis [56]. Being more convenient for large-scale production though, RT synthesis also shows some drawbacks like limited control of QDs shape and possible dissolving of the produced QDs due to the existence of polar solvents [65–68].

### 2.2. Structures of HP-QDs based composites and their fabrication strategies

**Scheme 2.**
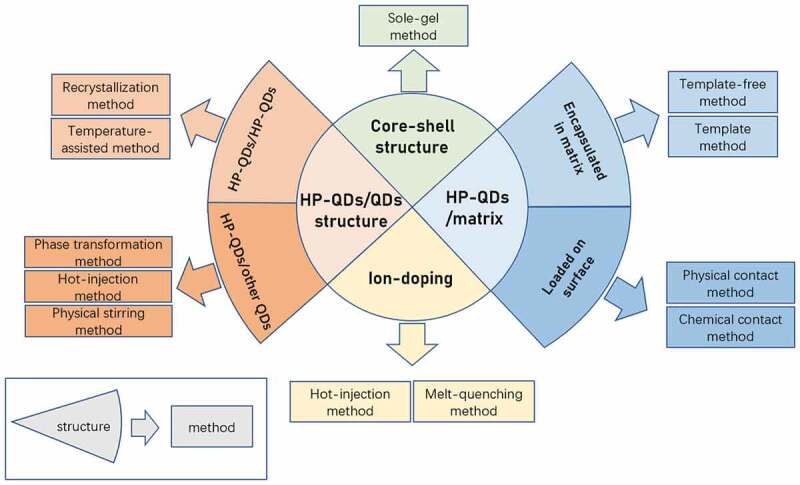
An overview of the synthesis methods for various structures of HP-QDs based composites.

The HP-QDs-based composites can be mainly divided into core-shell structure, HP-QDs/matrix structure, ion doping and HP-QDs/QDs structure. Here, the fabrication strategies of these four kinds of HP-QDs based composites are discussed separately (as shown in Scheme 2). It is worth noting that the difference between ‘core-shell structure’ and ‘HP-QDs/matrix structure’ is the number of HP-QDs. In ‘core-shell structure’, there are one or few HP-QDs are defined as ‘core’. However, there are large amounts of HP-QDs in the matrix in the structure of ‘HP-QDs/matrix’.

#### 2.2.1. Core-shell structure

Shell design is considered as a promising way to protect sensitive materials from degradation caused by environmental factors. By covering the QDs with a sturdy and inert shell, not only can the formed shell insulate the QDs from oxygen or water molecules but also this outside layer can passivate the surface of the HP-QDs by decreasing its high surface energy and increasing the energy barrier [53]. Oxide materials have been successfully applied as the shell material to form exquisite structure with great monodispersity. Here, taking examples of the most commonly used shell materials SiO_2_ [69] and TiO_2_ [70], several kinds of modulated sol-gel methods for the formation of the shell are discussed.

SiO_2_ has been utilized as shell material for QDs like CdSe [71] for a long history since 2006 due to its robustness and chemical stability. Commonly, as shell material, SiO_2_ was synthesized by direct hydrolysis method in QD solutions of water and ethanol using tetraethyl orthosilicate (TEOS, SiOC_2_H_5_) as silica precursor [72]. This method is called sol-gel method [73], and its chemical process could be divided into three steps [74]:

(1) $$-SiOC_{2}H_{5}-+H_{2}O\to -SiOH+C_{2}H_{5}OH$$

(2) $$-SiOH+SiOC_{2}H_{5}\to -SiOSi-+C_{2}H_{5}OH$$

(3) $$-SiOH+-SiOH\to -SiOSi-+H_{2}O$$

However, this traditional method is not completely suitable for HP-QDs due to the presence of water as media. To form a water-less reaction system, various kinds of modulated sol-gel methods have been carried out. In 2017, Hu et al. used 2-methoxyethanol to replace water molecules. In this work, the functional –OH group of 2-methoxyethanol helped to transfer TEOS to Si-O bonds and also passivate the surface of CsPbBr_3_ QDs to improve its water-resistance [74]. Another group led by Huang adopted orthosilicate (TMOS, Si(OCH_3_)_4_) as silica precursor and toluene as reactive solvent with a water content of only 0.0184% to encapsulate MAPbBr_3_-QDs (MA: CH_3_NH_3_) [75]. In this process, the hydrolysis rate is relatively fast (4 h) for the faster water consumption rate of TMOS [75]. Cai et al. instead utilized a heating treatment in the open air to attract water molecules from the air to initiate the hydrolysis. Adding the capping agents in the waterless solution before the synthesis of HP-QDs resulted in a better control of the QD size [76].

**Figure 1.**
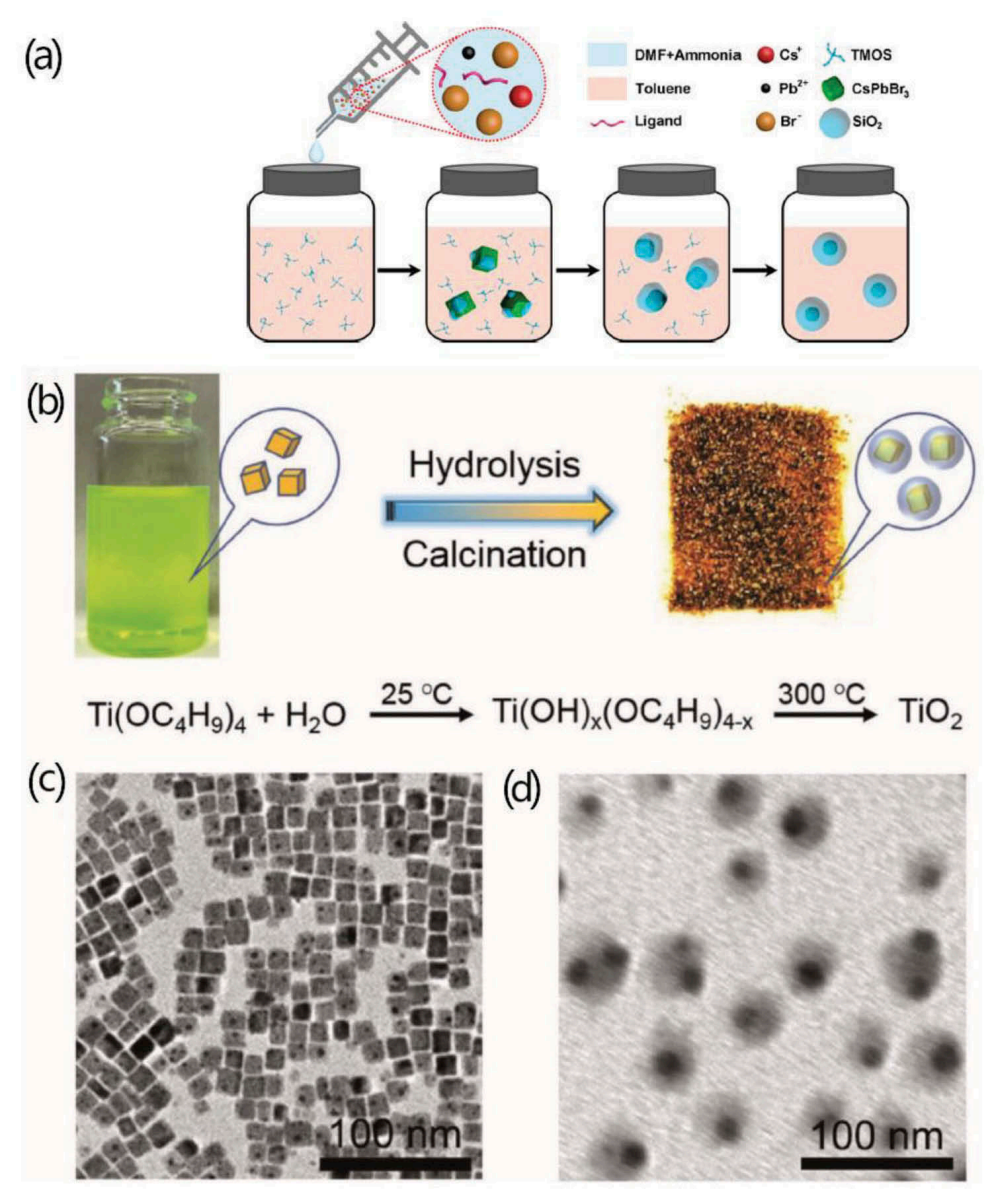
Schematic illustration of the synthesis strategies of (a) CsPbBr_3_ QD core/SiO_2_ shell structure. (b) CsPbBr_3_ QD core/TiO_2_ shell structure; transmission electron microscopy (TEM) images of (c) CsPbBr_3_ QDs (d) CsPbBr_3_ QD/TiO_2_ composites at single particle level. Produced with permission from [69]. © 2018 ACS Publications, and [70]. © 2018 Wiley.

In 2018, a modulated one-pot approach for HP-QDs/SiO_2_ composite at single-particle level was realized in a report by Zhong et al. using TMOS [69]. Both the perovskite QDs and the outer silica shell were synthesized in one-pot process at room temperature. In this process, at 30°C, the mixture of perovskite precursor including CsBr, PbBr_2_, oleic acid and oleylamine dissolved in dimethylformamide (DMF) was quickly injected into toluene solution of tetramethoxysilane (TMOS). The perovskite QDs were obtained immediately by recrystallization. Then, after two more hours under magnetic stirring, the CsPbBr_3_ QDs core (with average size of 10.5 nm)/SiO_2_ shell (with average thickness around 7.7 nm) structure was prepared (Figure 1a). In the presence of oleic acid and oleylamine, the firstly synthesized QDs could be well dispersed uniformly within the solution, while the silica oligomers produced by TMOS were gradually attracted to the QDs to form the shell [69]. Their group has also successfully synthesized a CsPbBr_3_ QDs/SiO_2_ Janus structure earlier in 2018 via a quick injection of deionized (DI) water into the mixture of TMOS and Cs_4_PbBr_6_ within hexane solvent [77]. In that process, the growth of SiO_2_ happened at the interface of hexane and water, and the Cs_4_PbBr_6_ was transformed to CsPbX_3_ QDs.

Another modulated sol-gel method, called hydrolysis-calcination method was successfully used for synthesizing HP-QDs/TiO_2_ core/shell structure with outstanding monodispersity by Li’s group [70]. The HI synthesized colloidal CsPbBr_3_ QDs kept in toluene solution at 25°C was added dropwise by the prepared titanium dioxide precursor titanium butoxide (TBOT) under stirring within 30% humidity, allowing the hydrolysis reaction to happen and form a complex compound of titanium (as shown in Figure 1b). At this step, large area of TiO_x_ matrix around the QDs was observed. Then, the solution system was heated to 300°C for calcination and removal of water, yielding well-dispersed core/shell structures with a high abundance of single-particle-core and about 5 nm TiO_2_ shell [70].

Thanks to the inert shell formed by oxides, the HP-QDs were fully coated with few oxygen or water molecule getting though, achieving long-tern stability against air. And the surface of QDs was passivated to reach much better water-resistance. Placing both the HP-QDs/SiO_2_ composites and pure HP-QDs within an environment of 75% humidity and exposed to air, the X-ray diffraction (XRD) peak of the composite only decreased slightly after 4 weeks while the pattern of pure QDs has been totally distorted within 3 days [69]. The CsPbBr_3_/TiO_2_ composite showed even better stability against water for maintaining great optical properties after 3 months within it, and even the size and morphology of the composite kept unchanged along this time. The stability against UV light was also tested, 75% PL intensity of the original value was kept after 24 h. Besides the outstanding enhancement in stability, the exquisite core/shell structure could help reduce anion-exchange effect to some extent since the QDs were insulated from each other, and TiO_2_ shell was also verified to promote charge carrier transfer [70].

#### 2.2.2. HP-QDs/matrix structure

##### 2.2.2.1. Encapsulated into material matrix

Without extreme high requirement for monodispersity, encapsulating a vast of HP-QDs into matrix of the coating materials is considered a more simple and efficient strategy to insulate and protect the HP-QDs, and thus is a more widely adopted method. Common materials utilized to encapsulate HP-QDs contain polymers, oxides, metallic compounds like CaF_2_ [78], ionic compounds like NaNO_3_ [79] and other organic materials like carboxybenzene [80]. And the synthesis strategies here are divided into template-free methods and template methods according to whether a template is adopted.

###### 2.2.2.1.1. Template-free methods

*Sol-gel method*. Sol-gel method is one kind of template-free method that is commonly used for synthesizing HP-QDs/Oxide matrix composites. Usually, the process could be divided into two parts: fabricate HP-QDs first, and then inject the oxide precursor into the HP-QDs solution system. After the mixing, the pre-synthesized HP-QDs and the precursor, which is the resource of oxide molecules, got in touch. Then, the precursor molecules went through a hydrolysis process to form oxide matrix around the QDs as protection. Similar to the synthesis of shell materials discussed before, the choices of precursor reagent and medium of the solution system are the key factors to the properties of resulted HP-QDs/SiO_2_ composites.

**Figure 2.**
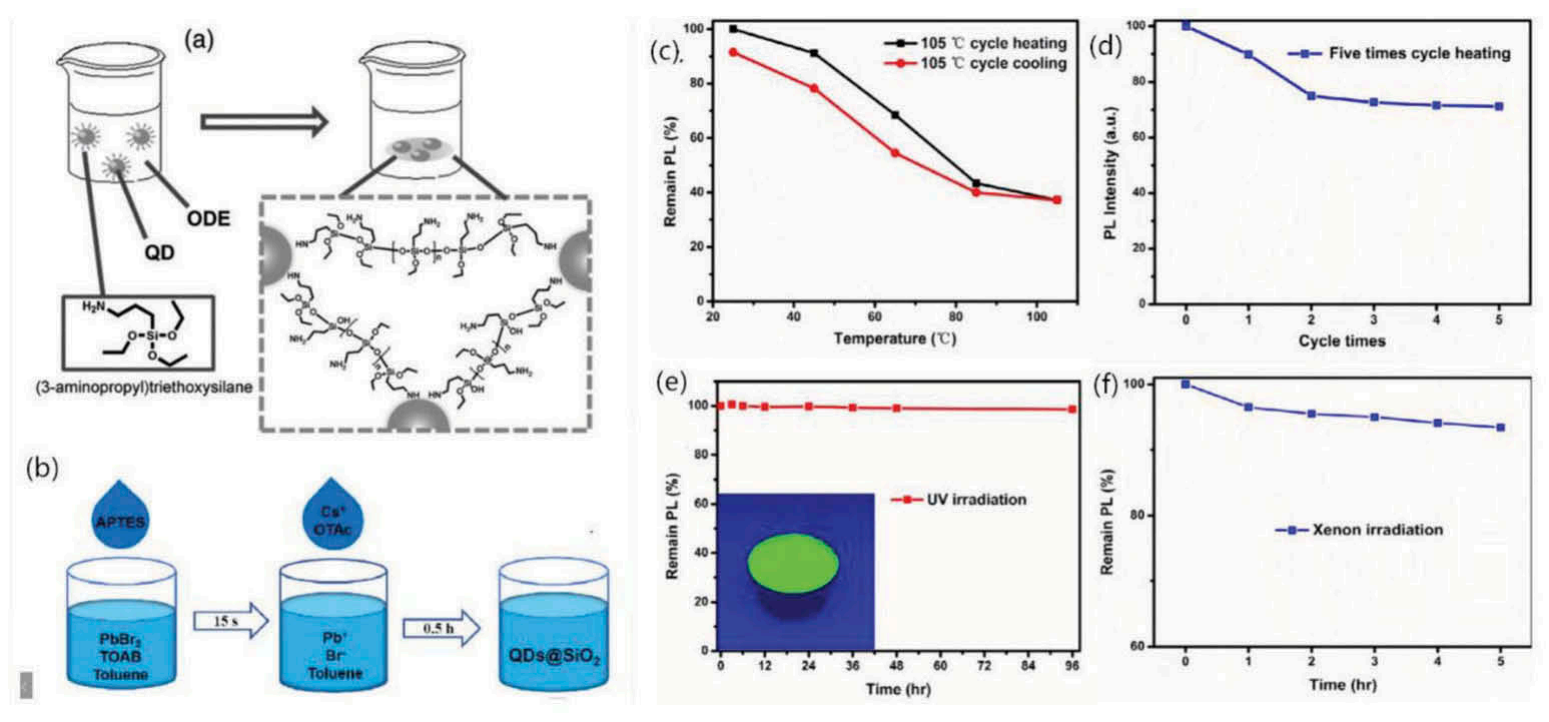
(a) Schematic illustration of synthesis strategy of QD/silica composites. (b) The simplified synthesis of CsPbBr3/SiO2 composite; PL intensity of CsPbBr_3_/SiO_2_ composite under (c-d) heat, (e) 365 nm UV light (f) xenon lamp irradiation. Produced with permission from [74]. © 2016 Wiley, and [82]. © 2019 ScienceDirect.

For silicon oxide, Sun et al. [74] applied (3-aminopropyl)triethoxysilane (APTES) to an organic cross-linked Si-O-Si silica matrix. In this work, non-aqueous solvent octa-decene (ODE) was used to mix the APTES and HP-QDs for a waterless system while the freely dispersed QDs were capped by the whole APTES molecules as a shell, indicating that the formation of silica matrix did not begin. Then, the solution system was exposed to the ambient air to allow the water molecules to be caught and initiate the hydrolysis process. As shown in Figure 2a, APTES here played important roles since not only it was hydrolysed to generate the Si-O-Si cross-linked silica structures over the QDs but also the surface of the QDs was passivated by its amino group to maintain the original optical and photoelectronic properties well [74].

On the basis of this APTES mechanism, in 2017, Sun’s group further developed their Si-O-Si network strategy and successfully embedded CsPbX_3_ QDs into organic silica gel matrix via a HI of APTES and perovskite precursor Cs-oleate by turns for excellent flexibility, transparecy and enhanced stability. And since the organic silica matrix was chemically bonded to the QDs, this composite structure could be well maintained without shrinking [81]. On the other path, in 2019, Cao et al. further simplified the traditional APTES method to realize that no complex operation other than stirring was needed during the whole process (Figure 2b). The as obtained CsPbBr_3_/organic silica matrix composite was then observed to exhibit enhanced photo- and chemical stability for a limited decrease of 5.7% and 1.4% in PL intensity after 30 days in air and 96 h under UV light (Figure 2c-f) [82].

Besides APTES for organic silica matrix, perhydropolysilazane (PHPS) [83] as precursor to form dense inorganic silica matrix with enhanced hardness and chemical stability, zirconium n-propoxide as precursor for ZrO_2_ matrix [84], Al-Si precursor for SiO_2_/Al_2_O_3_ binary matrix [85], polysilazane for SiN_x_/SiN_x_O_y_/SiO_y_ matrix structure [86] were also reported using similar sol-gel method.

*Atomic layer deposition*. Atomic layer deposition (ALD) was adopted to encapsulate QDs by AlO_x_ in 2017 [87]. ALD is a technique that can deposit materials layer by layer at single-atom level on the surface of the substrate [88]. The application of ALD on HP-QDs was firstly reported by Loiudice et al. They spin-coated CsPbX_3_ QD on a substrate and then deposited an amorphous alumina shell around the whole CsPbX_3_ QD layer. Optimal parameters of ALD were studied and the composites exhibited excellent stability against water and heat [87]. Metal oxides like AlO_x_ are also good candidates as encapsulating materials for their transparency and outstanding performance in protecting QDs from oxidative and other chemical factors due to their low ion diffusion rate. With this protection layer, the as prepared CsPbX_3_ QDs/AlO_x_ composites exhibited great stability in air (for 45 days), under irradiation (8 h), heat (200°C) and water (1 h after being immersed) [87].

*Crystallization method*. Crystallization method is commonly adopted for encapsulating HP-QDs in polymer matrix where the precursor of HP-QDs were added into the matrix. The precursor capped by the polymer structures would form QDs inside the matrix via crystallization process. Based on various specific operation methods, it could be further divided into simple blending [89,90], separate crystallization [91], swelling-deswelling [92] and melting-quenching with subsequent heat-treatment [24].

Early in 2015, Li et al. reported crystallization of organic halide perovskite quantum dots (OHP-QDs) inside polymer matrix via a simple and direct blending of perovskite precursor and polymer matrix followed by annealing. The composite structure MAPbBr_3_-QDs/4,4-bis(N-carbazolyl)-1,1-biphenyl (CBP) was successfully obtained [89]. Another group of Li et al. employed polyimide precursor dielectric (PIP) and obtained the PIP/MAPbBr_3_ QDs composites by so too. The as obtained composite thin film was pinhole-free and exhibited enhanced quantum efficiency [90]. However, the dispersity of QDs inside the polymer was relatively low by physical blending only. To enhance the dispersity, two developed strategies of crystallization called separate crystallization and swelling-deswelling were carried out.

**Figure 3.**
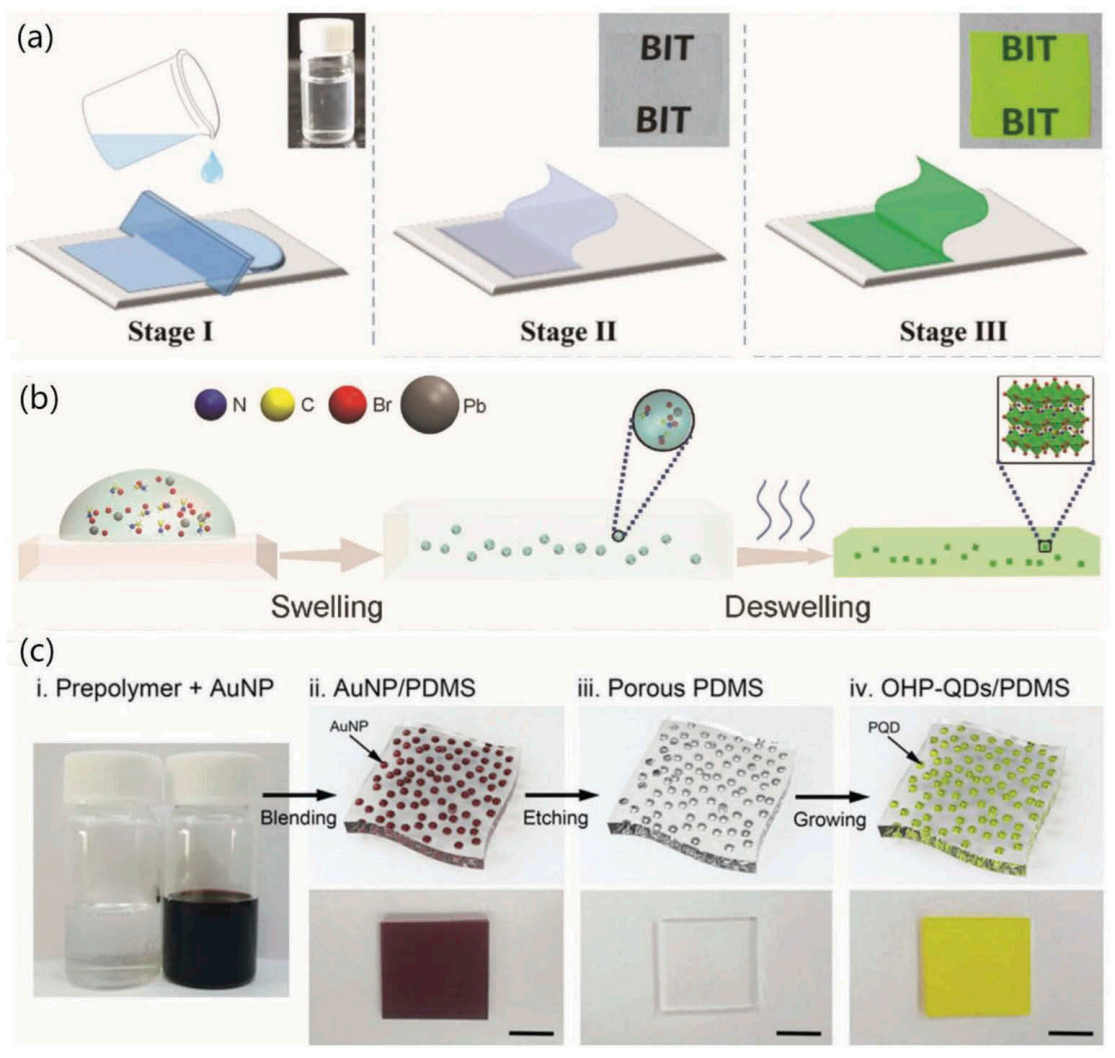
(a) Schematic illustration of MAPbBr_3_-QDs/PVDF synthesis via separate crystallization: Stage Ⅰ: mixture poured on the substrate; Stage II: evaporation of DMF; Stage III: removing the residual DMF. (b) Schematic illustration of the formation via Swelling-deswelling, and (c) via porous polymer template. Reproduced with permission from [91,92]. © 2016 Wiley, and [105]. © 2017 Royal Society of Chemistry.

Separate crystallization strategy (Figure 3a) was firstly presented by Zhou et al. in 2016 [91]. In their report, the precursor of MAPbBr_3_-QDs and precursor of polyvinylidene fluoride (PVDF) were all dissolved in DMF solvent uniformly. With the removing of DMF by vacuum pumping, PVDF crystals were formed first. As the concentration exceeded the limit, MAPbBr_3_ would finally be crystallized under the confinement of existed polymer matrix. The interactions between –CF_2_– group of the PVDF and MA^+^ component of MAPbBr_3_-QDs was also verified to play an important role of helping obtain uniform size and distribution during the crystallization of MAPbBr_3_ [91]. Similarly, by pouring the DMF solution of perovskite precursor and NaNO_3_ into poor solvent toluene, HP-QDs/NaNO_3_ composites could also be obtained via crystallization method [79].

Swelling-deswelling is another mechanism to obtain great monodispersity of HP-QDs inside the polymer matrix (Figure 3b). In 2016, Wang et al. reported this method and tried it on various polymer materials including commonly used polystyrene (PS), polycarbonate (PC), poly(methyl methacrylate) (PMMA) and many other polymers that swell in DMF, such as acrylonitrile butadiene styrene (ABS), cellulose acetate (CA) and polyvinyl chloride (PVC) [92]. Swelling occurred when the specific polymer was in the DMF solvent, the chains of polymer swelled and expanded and allow the perovskite precursors to be carried inside it uniformly. As the DMF solvent is gradually removed, nanosized HP-QDs were formed followed by the deswelling process where the polymer chains would shrink back to coherently encapsulate the HP-QDs inside. The as obtained MAPbBr_3_ QDs/polymer composites processed enhanced dispersion and stability against water and heat [92].

Melting-quenching with subsequent heat-treatment is always utilized for inorganic polymers including phospho-silicate glasses [24,93], tellurite-based glasses [94], borosilicate glasses [80,95] and boro-germanate glasses [80,96]. In this process, the properly designed glass matrix and the precursors of perovskite were firstly prepared and mixed together as powders. After melting at a high temperature, the precursors of perovskite were uniformly encapsulated by molten glass, and then went through self-crystallization to form HP-QDs/glass composites under a heat-treatment at a relatively low temperature [24]. The resulting HP-QDs/polymer composites exhibited excellent stability and preserved 100% of their PL quantum for 30 days in air and 85% PL quantum yield after 10 days in water [97].

*Electrospinning method*. Electrospinning method, a simple and low-cost technique, where fibers could be obtained from polymer solution under a strong electronic field, was also used to fabricate HP-QDs/polymer composites. In 2016, Wang et al. synthesize CsPbX_3_ QDs/PS composite in a fiber membrane structure using this method. The electrospinning solution was prepared by dissolving PS and CsPbX_3_ QDs in the toluene solvent added by conductivity-assisted DMF. The result showed that CsPbX_3_ QDs distributed inside the PS fiber with outstanding dispersion, remained optical properties and enhanced stability to water and UV light [98]. Moreover, instead of using pre-synthesized HP-QDs solution, Liao et al. slightly adjusted this strategy by blending solution of perovskite precursors with the polymer as the electrospinning solution, and realized tunable optical properties by controlling the composition of the perovskite precursor [99].

*Monomer-polymerization method*. Forming HP-QD/monomer structure first and then polymerizing the monomers to obtain polymer matrix is another strategy. In 2018, Xin et al. adopted this technique using monomers to fabricate HP-QDs/polymer composites with high stability and flexibility. They transported perovskite precursor solution into bulk monomers of styrene, then UV-light or thermal process was carried out for its polymerization [100]. Similarly, other polymer materials, such as epoxy resin also can be used to develop HP-QDs/polymer composites using this monomer-polymerization method [97].

###### 2.2.2.1.2. Template methods

The template method here is a method that precursors of perovskite were always added into the pre-synthesized mesoporous template, resulting in confined growth of HP-QDs within the holes wrapped by the matrix. This template structure is also expected to hinder interactions among different kinds of HP-QDs, thereby inhibiting anion exchange on optical properties. Thus, various mechanism of carrying perovskite precursors uniformly inside the pores were reported to realize a better monodispersity including physical stirring [101], capillary force [102] and recrystallization [103].

**Figure 4.**
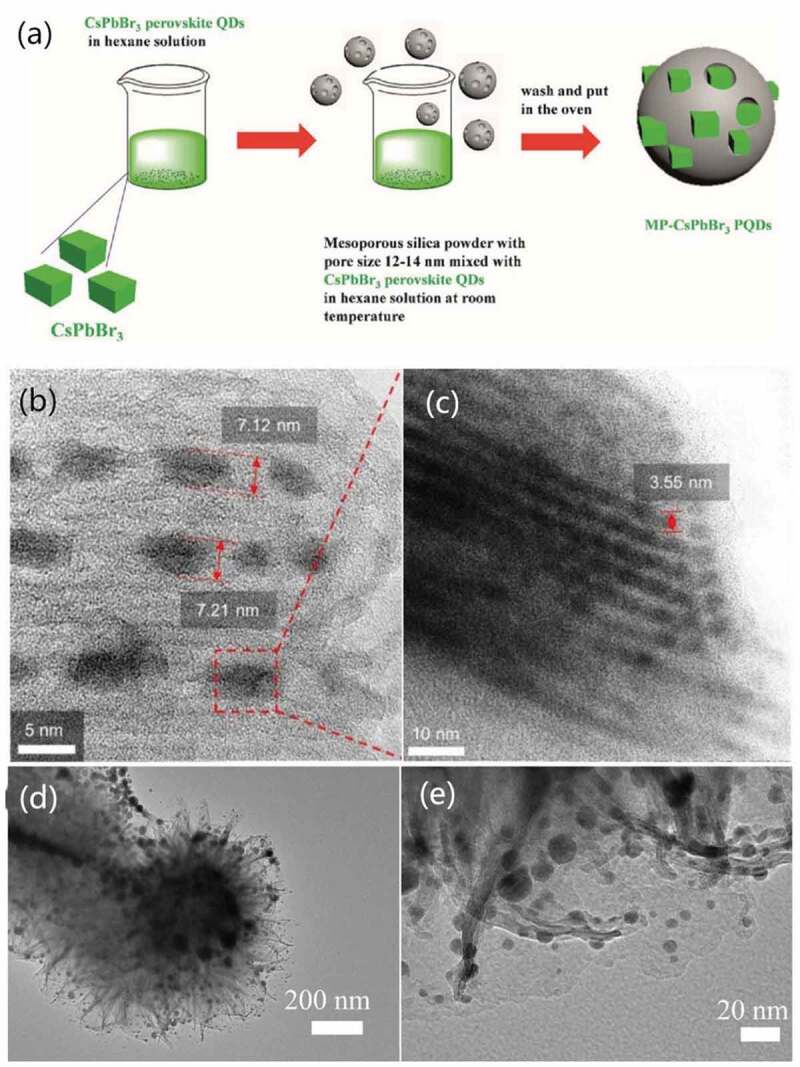
(a) Schematic illustration of HP-QDs/MSP synthesis via physical stirring. (b-c) TEM images of HP-QDs/MSP obtained via capillary force. (d-e) TEM images of HP-QDs/MSP obtained via recrystallization. Reproduced with permission from [101]. © 2016 Wiley, and [102]. ©2016 ACS publication, and [103]. © 2018 ACS publication.

Mesoporous silica powder (MSP) was always used as template matrix, here as an example to state these mechanisms. In 2016, simple physical stirring (Figure 4a) was used in the report by Wang et al. They mixed the MSP and precursor of inorganic CsPbX_3_ QDs in non-polar solvent hexane followed by continuous stirring and by so obtained the silica-wrapped CsPbX_3_ QDs structure with various pore size [101]. Another team of Malgras et al. dissolved the precursor of organic MAPbBr_x_I_3-x_ QDs in N,N-dimethylformamide (DMF) and added it dropwise into the dried MSP, utilizing capillary force from the difference in surface tension between DMF and silica to bring HP-QDs precursor into the pores uniformly (Figure 4b,c). The composites exhibited excellent thermal and photostability [102]. Recrystallization mechanism was later adopted by Zhao et al. in 2018 using magnesium silicate hollow spheres (MSHS) as the template [103]. In their work, a MAPbX_3_ QDs/MSHS composite (Figure 4d,e) was synthesized by dropping perovskite precursor which was dissolved in good solvent DMF into toluene solution of MSHS. Tunability of emission from blue to red and outstanding thermal and photostability were verified [103].

Immersion is a more simple and convenient method for templates other than powders where the well-designed template was directly immersed into the solution of perovskite precursor. In 2017, Demchyshyn et al. chose nanoporous alumina scaffold as template to directly synthesize HP-QDs within specific format [104]. In their work, firstly the mesoporous aluminum oxide nanotubes were prepared via evaporating aluminum on glass substrate, followed by anodizing with specific voltage to form nanosized pores. After cleaning processes, the alumina nanotubes were infiltrated into perovskite precursor solution to confine the growth of HP-QDs carried inside the pores [104]. Similarly, porous polymer material obtained by preparing polydimethylsiloxane (PDMS) film with many size-controllable gold nanoparticles (AuNPs) inside and then remove the AuNPs [105] has also been reported to obtain composites with HP-QDs via immersion method (Figure 3c).

Using template-methods to form HP-QDs-based composites could help control the emission wavelength of HP-QDs and enhance their stability at the same time [106]. Compared with simple stirring method and recrystallization method that lead to random distribution of QDs, capillary force method or immersion method with well-designed templates could firstly better encapsulate the ordered HP-QDs inside the matrix for enhanced stability, and secondly improve the monodispersity of HP-QDs within the pores. The monodispersity could contribute to better prevent on anion-exchange effect of MAPbX_3_ with tunable ratio of the X components for tunable emission peak. It was verified that the obtained great tunable properties of HP-QDs also benefits from the controllable QDs size by the controlled pore size of the templates, since not only did the template act as isolating layer, it also help confine the growth of QDs to reduce its structural disorder [102].

Besides these strategies, CsPbBr_3_ QDs/ethylene vinyl acetate (EVA) composite with long-term stability and great flexibility was obtained by Li et al. via one-pot RT-synthesis through recrystallization of CsPbBr_3_ QDs and dissolution of EVA [107]. And for specific application, Huang’s team in 2018 and Tana et al. in 2019 adopted molecularly imprinted polymers (MIPs) for sensitive detection [108,109], and Xuan et al. employed hydrophobic porous polymer frameworks (SHFW) to obtain HP-QDs-based composite with extraordinary water-resistant performance [110].

##### 2.2.2.2. Loading on materials surface

Loading HP-QDs on the surface of other materials is another efficient way to form composites and usually leads to nanojunctions. In this part, synthesis methods for composite structures differ from physical contact to chemical bonding would be discussed separately.

###### 2.2.2.2.1. Physical contact

Physical contact structure is commonly obtained via relatively direct and simple physical methods including spin-coating and physical blending. Using this method, the utilization of metal oxides including TiO_2_ [34], Al_2_O_3_, ZrO_2_ and SnO_2_ [106] were studied at 2009 and 2012 by Kojima et al. In their work, prepared porous Al_2_O_3_ paste and the perovskite precursor solution (MABr and PbBr_2_ in DMF) were successively spin-coated on a glass substrate. Al_2_O_3_/MAPbBr_3_ nanocomposites were obtained via self-organization of HP-QDs separately on the surface of the Al_2_O_3_ spheres. The ZrO_2_/MAPbBr_3_ composites, SnO_2_/MAPbBr_3_ composites, TiO_2_/MAPbX_3_ composites were obtained via a similar process. Enhanced optical properties of these heterojunctions were also reported which benefit from the greater energy band gap and ionization potential of Al_2_O_3_ and ZrO_2_ to intense emission [106], and lower conductive level of TiO_2_ and SnO_2_ to speed up the electron injection [111].

Blending Al_2_O_3_ nanocrystals and the perovskite precursor together first and then spin-coated it on the substrate, Longo et al. obtained and further studied the Al_2_O_3_/MAPbBr_3_ nanocomposites thin film. In this work, they stated the assistance of aluminum oxides in confining perovskite into nanoscale by comparing results with different amount of Al_2_O_3_ [112].

Spin-coating pre-synthesized HP-QDs on the surface is another convenient physical method. In 2017, MAPbI_3_ solution was spin-coated over the TiO_2_ nanotubes (NTs) to obtain MAPbI_3_/TiO_2_ NTs composites by Zheng et al., and enhanced stability against moisture/heat and the improved responsivity in photodetector application area were verified [113]. Lu et al. spin-coated CsPbX_3_ QDs on the 3D radial junction over a silica nanowire structure [114]. In 2019, Zhao et al. prepared a composite structure of Ag/CsPbBr_3_-QDs/g-C_3_N_4_ (CN) via spin-coating, where the HP-QDs were uniformly distributed on CN layer, followed by a layer of Ag spin-coated on the above for photocatalyst [115].

Besides these physical methods, one-pot synthesis for HP-QDs/nanosheet has also been reported with graphene oxide (GO) as matrix material [116]. In that work, the graphene oxide and PbBr_2_ were blended in DMF together followed by quick injection of the perovskite precursor Cs-oleate solution. The as obtained CsPbBr_3_ QD/GO composite, with HP-QDs uniformly distributed on the GO sheet, exhibited great electron consumption rate and potential in photocatalysis applications [116].

###### 2.2.2.2.2. Chemical bonding

Chemical bonding structure here could be obtained via two main strategies: linker molecules and ion exchange reaction. For using linker molecules, Zhou et al. in 2017 reported a TiO_2_/HP-QDs composite using bifunctional linker molecule 3 mercaptopropionic acid (MPA) to improve the electron transfer rate of the nanojunction [117]. TiO_2_ paste, pure MPA and CsPbBr_3_ QDs solution were spin-coated in turn on the substrate, where the thiol groups of MPA interacted with CsPbBr_3_ QDs with its carboxylic groups reacting with hydroxyl group of TiO_2_ nanoparticles. The TiO_2_/MPA/CsPbBr_3_-QDs composites were obtained with enhanced electron transfer rate.

**Figure 5.**
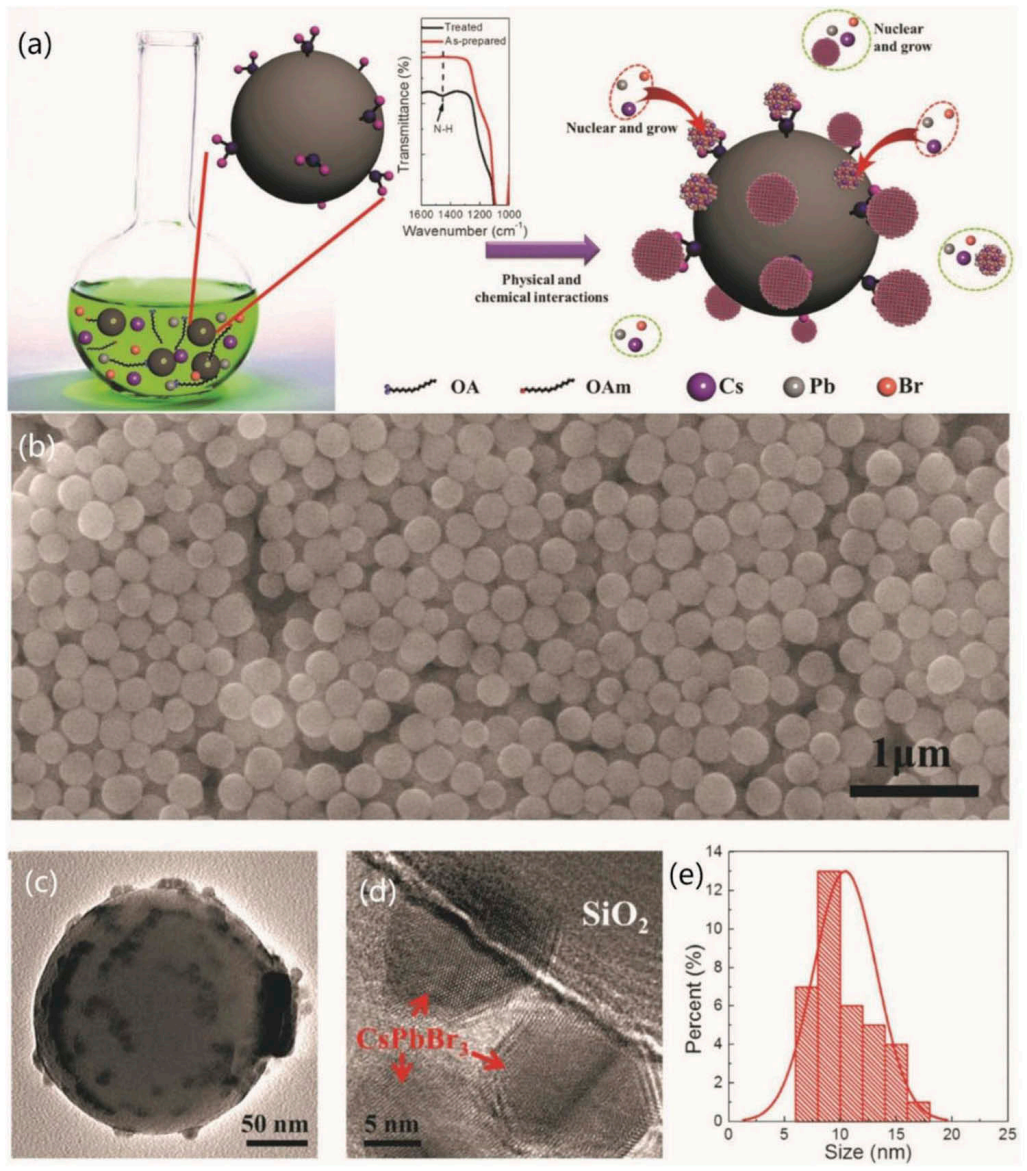
(a) Schematic illustration of the a-SiO_2_/HP-QDs synthesis. (b-d) TEM images of the a-SiO_2_/HP-QDs composite. (e) Size distribution of the loaded HP-QDs. Reproduced with permission from [118]. © 2017 Wiley.

Silica oxide, as another versatile oxide material, has also been explored in this area using monodisperse aminated SiO_2_ (A-SiO_2_) spheres (Figure 5) [118]. In this work, they blended A-SiO_2_ spheres, perovskite precursor, surfactant together in ODE followed by heat-treatment. During this process, amination played the important role of stimulating the adhesion of HP-QDs through chemical interaction, and the HP-QDs would grow on or attach to the spheres. These A-SiO_2_/HP-QDs composites were verified to exhibit outstanding stability for performing little PL degradation after 40 days, and only 20% degradation after 108 h under UV light [118].

Ion-exchange reaction method was utilized for CsPbBr_3_@NH_4_Br composites by Lou et al. They added excess NH_4_Br into a toluene solution of CsPbCl_3_ nanoparticles where the CsPbCl_3_ would be transformed into CsPbBr_3_ by anion-exchange effect when getting close to the NH_4_Br molecules. Then, with the replacement of Cl^−^ to Br^−^, the HP-QDs were chemically attached to the NH_4_Br and thus formed tense composites. The composites showed enhanced thermal stability than pure QDs. And the [NH_4_]^+^ group could help stabilize the colloidal structure of HP-QDs and by so improve their stability against polar solvent as water [119].

#### 2.2.3. Ion doping

Ion doping is a promising strategy in adjusting the electronic and optical properties of QDs [120]. For HP-QDs, it was verified that introducing metal ions like Mn^2+^ [121–124], Sn^2+^ [121,125], Ce^3+^ [126], Eu^3+^ [127] into the perovskite lattice of CsPbX_3_ QD could help reach tunable energy gap [123], enhanced thermal stability [121] and improved PL efficiency [126]. The fabrication strategies of ion doping contains hot-injection and melt-quenching.

**Figure 6.**
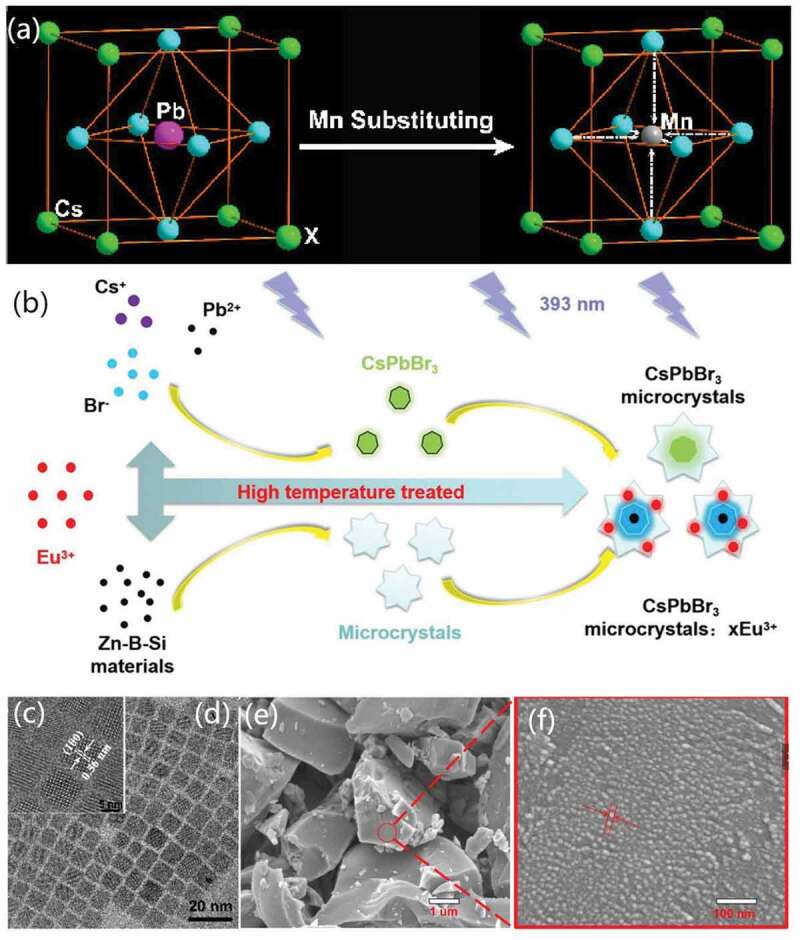
Schematic illustrations of (a) the structures of crystal lattice of perovskite CsPbX_3_ before and after Mn^2+^doping; (b) the formation of CsPbBr_3_ and Eu^3+^:CsPbBr_3_ QDs via melt-quenching. (c-d) TEM images of Mn^2+^:CsPbX_3_ QDs prepared via HI. (e-f) TEM images of Eu^3+^:CsPbBr_3_ QDs prepared via melt-quenching. Reproduced with permission from [121]. © 2017 ACS publications, and [127]. © 2019 ScienceDirect.

Hot-injection method was used to add the precursor of ions into the PbX_3_ solution followed by HI of Cs precursor. In 2016, the synthesis of Mn^2+^:CsPbX_3_ QDs composites was realized by this approach using MnX_2_ as the ion-precursor, where tunable band gap and mechanism were studied [123,124]. On this basis, by slightly modifying the Mn/Pb ratio in the process, Zou et al. further studied the Mn^2+^:CsPbX_3_ QDs composites and firstly stated its enhancement in stability against high temperature and ambient air. This was attributed to the reduced ionic radius of metal ions than the replaced Pb^2+^ in the lattice that leads to higher formation energy which would radically improve the thermal stability of HP-QDs (Figure 6a). In this work, the similar applications of Cd^2+^, Co^2+^, Zn^2+^, Sr^2+^ and Sn^2+^ ions in doping CsPbBr_3_ QDs were also explored [121]. Ce^3+^:CsPbBr_3_ QDs composite was synthesized by this method too in 2018, where the CeBr_3_ was hot-injected first into the PbX_3_ solution system followed by the halide precursor, exhibiting enhanced PL quantum yield [126].

Melt-quenching was used in 2019 for Sn^2+^ and Eu^3+^ doping by embedding HP-QDs inside borosilicate glass matrix [128]. Wu et al. reported Eu^3+^:CsPbBr_3_ QDs composites synthesized via blending Eu_2_O_3_, precursors of glass and precursors of CsPbBr_3_ together with proper ratio followed by heat treatment and cooling (Figure 6b) [127]. Sn^2+^:CsPbBr_3_ QDs composite was obtained similarly using SnBr_2_ instead as the source of Sn^2+^. This method promised enhanced stability against heat and open air due to the protection of glass in the synthesis process [125].

Ion-doped HP-QDs were combined with other compositing strategies mentioned above for enhanced stability, including encapsulating Mn^2+^: CsPbX_3_ QDs in polymer (epoxy resin) matrix [129], in oxide (SAM) matrix [130], in PMMA matrix and silica shell [131]. Ion doping for OHP-QDs using Cs to obtain organic-inorganic hybrid HP-QDs with improved optical properties and stability have also been studied [132,133].

#### 2.2.4. HP-QDs/QDs composite

**Figure 7.**
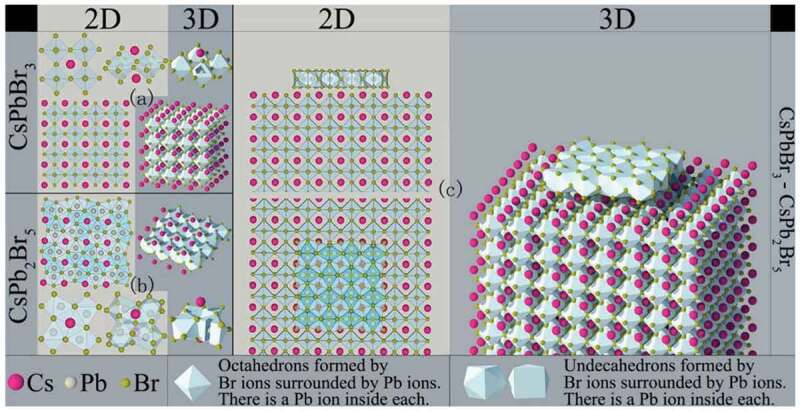
Crystal structures of (a) CsPbBr_3_, (b) CsPb_2_Br_5_ and (c) dual-phase CsPbBr_3_/CsPb_2_Br_5_ composite. Reproduced with permission from [134]. © 2018 ScienceDirect.

HP-QDs/HP-QDs composites, also called dual-phase HP-QDs composites, exhibited enhanced current efficiency, ionic conductivity, structural stability and emission lifetime [134,135]. The crystal structure of dual-phase CsPbBr_3_/CsPb_2_Br_5_ composite is shown in Figure 7. Its synthesis strategies include temperature-assisted methods and saturated recrystallization methods.

Temperature-assisted method was studied in 2016 by Zhang et al. [135]. In their report, the phase transition of HP-QDs at specific temperature was stated and dual-phase CsPbBr_3_/CsPb_2_Br_5_ composite was obtained via synthesizing CsPbBr_3_ QDs first in low temperature (100°C) followed by heat-treatment to 130°C, with excess PbBr_2_ as resources. Similarly, Song’s group synthesized CsPbBr_3_/CsPb_2_Br_5_ composite via a traditional hot-injection process (190°C) only with more PbBr_2_ in the solution [134].

Saturated recrystallization method was always utilized to obtain CsPbBr_3_/Cs_4_PbBr_6_ composites with various ratios by adjusting the ratio of Cs: Pb: Br in the reaction system. When the solution was Cs-rich or Br-rich, the main product would be Cs_4_PbBr_6_, which could be transferred into CsPbBr_3_ via reacting with excess PbBr_2_. On the contrary, when solution was Pb-rich, CsPbBr_3_ would be the main product [136,137]. On the basis of this mechanism, Li et al. synthesized CsPbBr_3_/Cs_4_PbBr_6_ composites by injecting precursor into antisolvent TEOS [138]. APTES has also been used here for binary protection of CsPbBr_3_@Cs_4_PbBr_6_/SiO_2_ structure [139]. Lou et al. obtained the composites via adding Br^−^ into saturated solution of PbBr_2_ and CsBr_2_ in 2019 [140]. Comparing with temperature-assisted method, this method is simpler and thus better for large-scale production.

Composites of HP-QDs and other QDs were also reported, such as CsPbBr_3_/Rb_4_PbBr_6_ QDs synthesized via phase transformation [141], CsPbBr_3_/PbSe nanocomposites with modified structure obtained via facile hot-injection synthesis [142] and CsPbBr_3_/ZnS QDs prepared by adding zinc precursor and sulfur precursor into the HP-QDs solution followed by physical stirring [143].

## 3. Applications of HP-QDs based composites

Based on different structures mentioned above, HP-QDs based composites have enhanced performance than pure HP-QDs in various applications, including white light-emitting diodes, photoemission, detector, photocatalyst, photovoltaic and memristor.

### 3.1. White light-emitting diodes

HP-QDs have been widely applied in white light-emitting diodes (WLED) as phosphor for their unique optical properties including high PL quantum yield and narrow bandwidth [53,144]. For HP-QDs based composites, the particular structure encapsulating HP-QDs within material matrix exhibited an even better performance in WLED since the matrix could not only improve the stability and working lifetime of HP-QDs in an open environment but also prevent anion exchange among different kinds of HP-QDs in the mixture [53].

**Figure 8.**
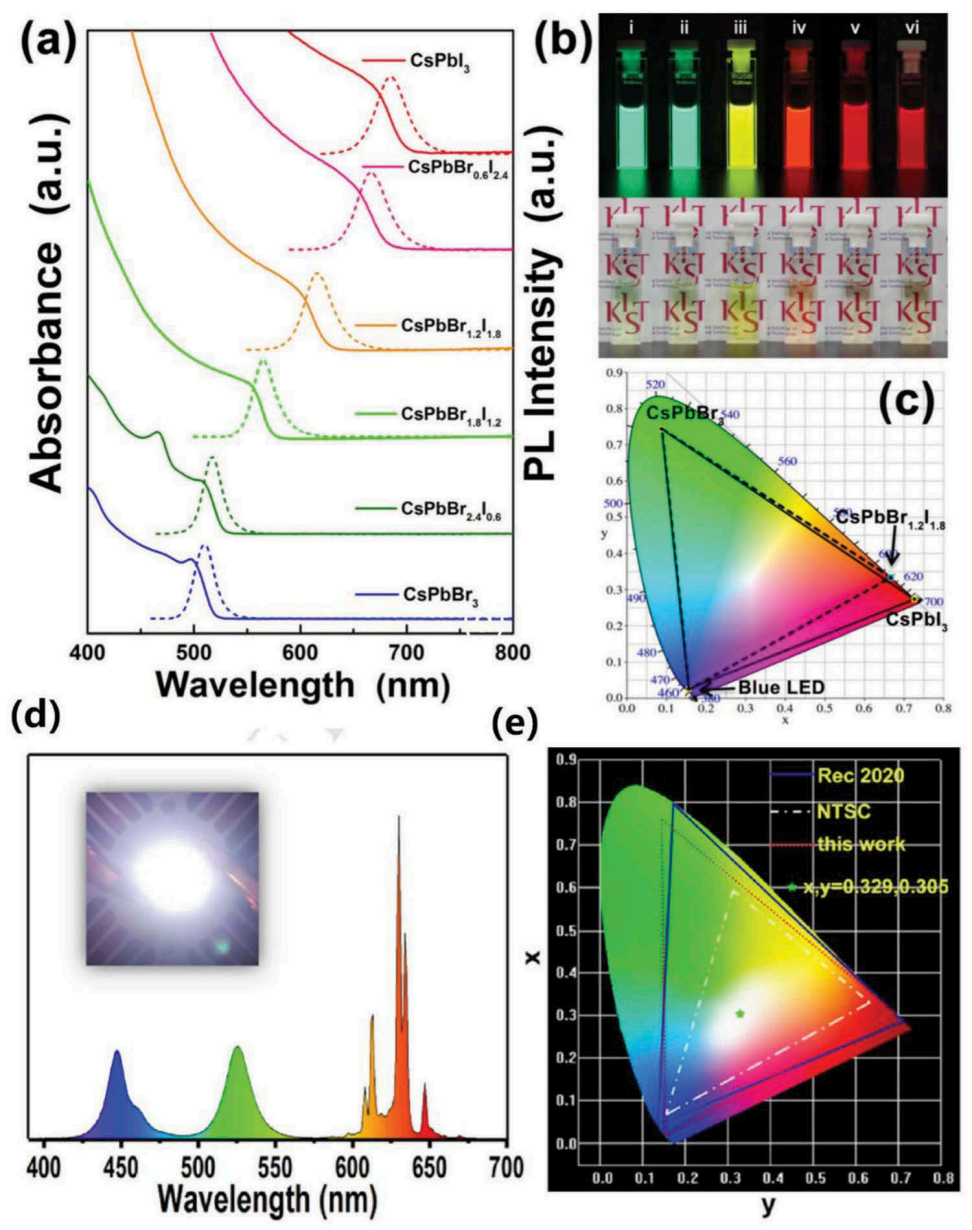
(a) PL spectra of CsPb(Br/I)_3_ QDs with varying Br/I ratio. (b) Picture of CsPb(Br/I)_3_ solution under UV light (top) and normal white light (bottom). (c) CIE color coordinates of CsPbBr_3_, CsPbBr_1,2_I_1.8_ and CsPbI_3_ separately. (d) Electroluminescence spectrum of CsPbBr_3_ QDs/SHFW composites, K_2_SiF_6_:Mn^4+^ and blue LED chip. (e) CIE color coordinate of CsPbBr_3_ QDs/SHFW/blue chip WLED. Reproduced with permission from [83]. © 2018 ScienceDirect, and [110]. © 2019 ACS Publications.

WLEDs are commonly obtained via combining green phosphor and red phosphor together on the blue LED chip. Various merits such as high color rendering index (CRI), high luminous efficiency (LE) and color coordinate of the Commission International de L’Eclairage (CIE) closer to the standard (0.33, 0.33) are required in WLED application. Since many review papers published recently had comprehensively listed the materials and parameters for HP-QD/matrix’s application in WLED [53,54], here we just stated them briefly. In some works, two types of HP-QDs based composites were blended together as the green/red phosphors (Figure 8a-c) [78,81,83,101,103,145–147]. For example, Sun et al. employed CsPbBr_3_ QDs/SiO_2_ composite as the green part and CsPb(Br/I)_3_ QDs/SiO_2_ composite as the red part and the as obtained WLED performed (0.33, 0.33) of CIE and LE of 61.2 lm/W [74]. Some other works adopted other materials such as CaAlSiN_3_ [82], CdSe [77], (Sr, Ca)AlSiN_3_:Eu^2+^ [107], CaSrAlN_3_:Eu^2+^ [125,148], K_2_SiF_6_:Mn^4+^ [86,91,110] as the red part or YAG:Ce^3+^ [129,130,149] as green part to be blended with HP-QDs-based composites (Figure 8d,e). CsPbBr_3_/SiO_2_ composite with CaAlSiN_3_,for example, was applied as green/red phosphor in Cao’s work and color coordinate of (0.3255, 0.3321) with 58.9 lm/W efficiency was obtained [82].

### 3.2. Photoemission

**Figure 9.**
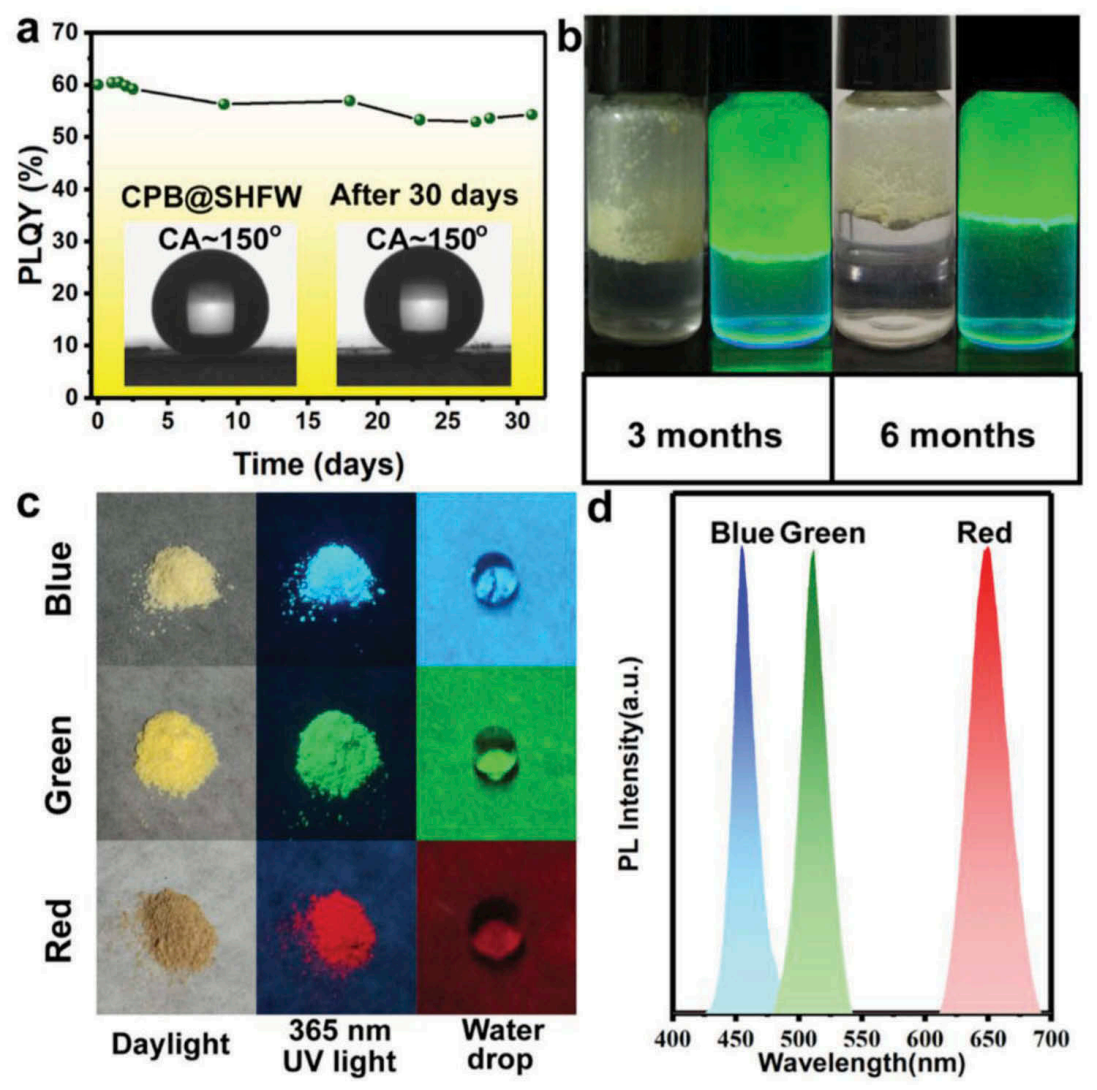
(a) Stability of PL quantum yield of CsPbBr3 QDs/SHFW in water. (b) Photos of CsPbBr_3_ QDs/SHFW in water after 3 months and 6 months under UV light (right) and normal white light (left). (c) Photos of CsPbBr_3_ QDs/SHFW in white light, UV light and under water drop. (d) PL spectra of CsPbBr_3_ QDs/SHFW composites. Reproduced with permission from [110]. © 2019 ACS Publications.

Pure HP-QDs have been widely applied in photoemission due to their outstanding electrical, optical properties. And with great performance as high PL value and external quantum eﬃciency (EQE), HP-QDs have been reported as promising candidate for photoemission devices as LED and laser [36,150,151]. For the composite structure of HP-QDs embedded in matrix, a good protection layer as silicon or polymer could efficiently enhance its stability against environmental factors, enabling the material to work within water, solar solvent or open air, the adoption of template in the fabrication process could also help maintain a smaller size of HP-QDs [99]. Thus, these merits make HP-QDs based composites great candidates for luminescent ink [99]. The application in light-emitting diodes of HP-QDs/oxide matrix [84,152] and HP-QDs/polymer matrix were studied and high PL emission was well maintained after treated with water (Figure 9a-d) [110], heat or UV light [84,99,152], high color purity via narrow FWHW (of 25 nm) [83,85], high LE (around 80 lm/W) [85], flexibility [90] were successfully obtained. In addition, for the strong scattering properties and greater optical gain, HP-QDs/SiO_2_ [118], HP-QDs/glass [94] composites had also been applied in random laser emission with obviously decreased threshold (by 50%) and enhanced efficiency (388%) [75].

The structure of HP-QDs loaded on the surface of oxides and the HP-QDs/HP-QDs composites were also applied in photoemission with improved performance due to their heterojunction structures. For HP-QDs/oxide, the greater energy band gap and ionization potential of Al_2_O_3_ and ZrO_2_ could help prevent electron injection and lead to intense emission [106,112]. For dual face HP-QDs/HP-QDs, the introduced lead-rich CsPb_2_Br_5_ QDs were found to help minimize free exciton emission and improve the ionic conductivity of pure CsPbBr_3_ QDs to reach an outstanding performance in LED with increased lifetime, EQE around 2.21% [135], narrower FWHM (19 nm) [138].

Ion-doping, another type of compositing, was verified to enhance the thermal stability of HP-QDs via enhancing the formation energy [121], and improve their PL efficiency and intensity [127] by modulating the PL kinetics, where EQE of 4.4% was achieved [126]. Besides, by introducing ions, the toxicity of Pb^2+^ could be reduced since some of them were replaced, and the emission peak position of HP-QDs could be adjusted by controlling the concentration of ions [153]. In the report of Eu^3+^:CsPbBr_3_ QDs by Wu et al., partial Eu^3+^ replaced Pb^2+^, leading to a blue shift of the peak, while others exhibited red light emission [154]. With the increasing concentration of Eu^3+^, more of them entered the cell structure of HP-QDs, broadened emission peak and adjusted light from green to blue and then red were achieved [127].

### 3.3. Sensor

**Figure 10.**
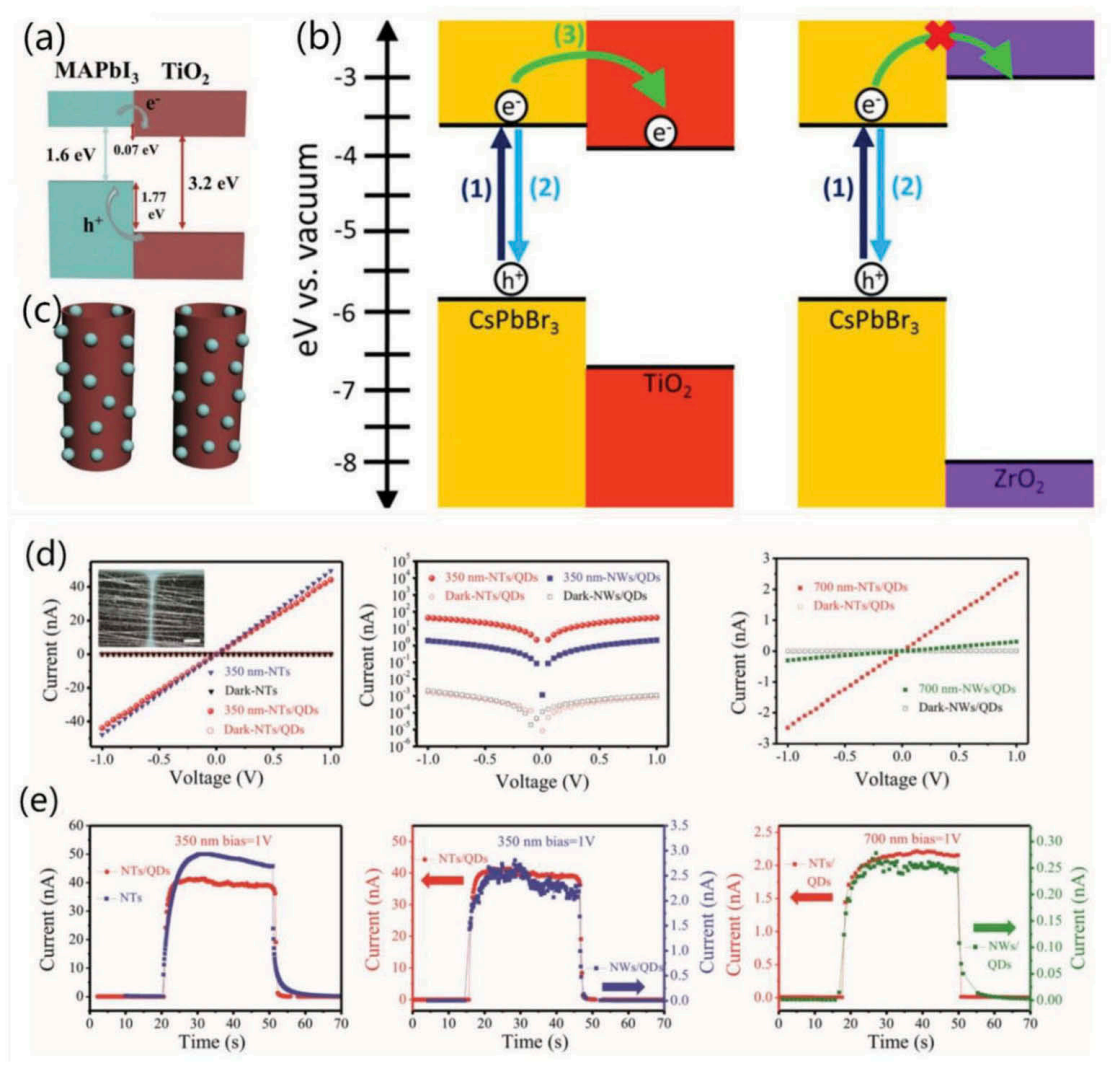
Energy level diagram of (a) MAPbI_3_/TiO_2,_ (b) CsPbBr_3_/TiO_2_ and CsPbBr_3_/ZrO_2_. (c) Schematic illustration of MAPbI_3_/TiO_2_ NTs composites. (d) I–V curves and (e) I-t curves of pure TiO_2_ NTs, MAPbI_3_/TiO_2_ NTs and MAPbI_3_/TiO_2_ NWs under darkness, 350 nm light, 700 nm light. Reproduced with permission from [113]. © 2017 Wiley, and [157]. © 2018 ACS Publications.

Optical sensing devices require a wide spectral response and high responsivity. Thus, semiconductor heterojunction with high charge separation and transport efficiency would be ideal candidate [113]. For the heterojunction of HP-QDs loaded on oxides’ surface, Al_2_O_3_ and ZrO_2_ were adopted in photoemission for their greater energy band gap, while on the contrary, possessing conductive level below the HP-QDs, TiO_2_, ZnO and SnO_2_ would easily permit electron injection (Figure 10a,b). Hence, the difference in band gap leads to efficient charge separation while the easy electron injection means fast electron transfer from HP-QDs to the oxide which ensures higher responsivity [117,155,156]. With these merits, the HP-QDs/TiO_2_ composite was applied as a great sensitizer where TiO_2_ play the role of n-type semiconductor [111,157–159].

For titanium, Zheng et al. decorated MAPbI_3_ QDs on TiO_2_ NTs to form heterojunctions with physical contact (Figure 10c). In their work, the MAPbI_3_/TiO_2_ NTs composites not only maintained the pure titanium’s absorption of UV light but also significantly improved the response performance in visible light for a broadened detection range. Also, the composites were verified to be more tolerant to moist air (72 h) and heat (100°C), and exhibited great flexibility and transparency (85%) [113]. HP-QDs/TiO_2_ composite with chemical contact using linker molecule MPA was reported by Zhou et al. They found that the composite with MPA showed much faster electron transfer from QDs to TiO_2_ (40 ns) than simple physical contact composite (290 ns). Thus, the responsivity was significantly enhanced from 2.2 AW^−1^ to 24.5 AW^−1^. It is also proved that the linker molecule could help attach more HP-QDs on the surface of TiO_2_ for higher efficiency [117,160]. Besides, MAPbBr_3_/TiO_2_ composites [111] and MAPbI_3_/TiO_2_ nanowires (NWs) [113], were all reported for the application in photodetector, reaching broadband detection (UV to entire visible range) and high electron injection efficiency (near 99%, Figure 10 d,e) [161].

HP-QDs/polymer composites have been applied in chemical sensors too. Wang et al. used PS fiber membrane to both improve the stability of HP-QDs and enhance the surface area for sensing Rhodamine 6 G [98]. And special molecularly imprinted polymer was adopted for its unique chemical properties as a recognition system for the HP-QDs to achieve high sensitivity and specificity in sensing phoxim [108].

### 3.4. Photocatalyst and photovoltaic

Photocatalyst for CO_2_ reduction, water splitting or degradation of organic compounds have attracted great attention as a promising strategy for solar-chemical conversion. Various materials have been applied for high efficiency, high selectivity and stable photocatalyst that could utilized visible light [162,163]. Processing a wide absorption range for visible light and long carrier diffusion length, HP-QDs could be ideal candidates for photocatalyst only if their poor chemical stability could be improved [164–166].

**Figure 11.**
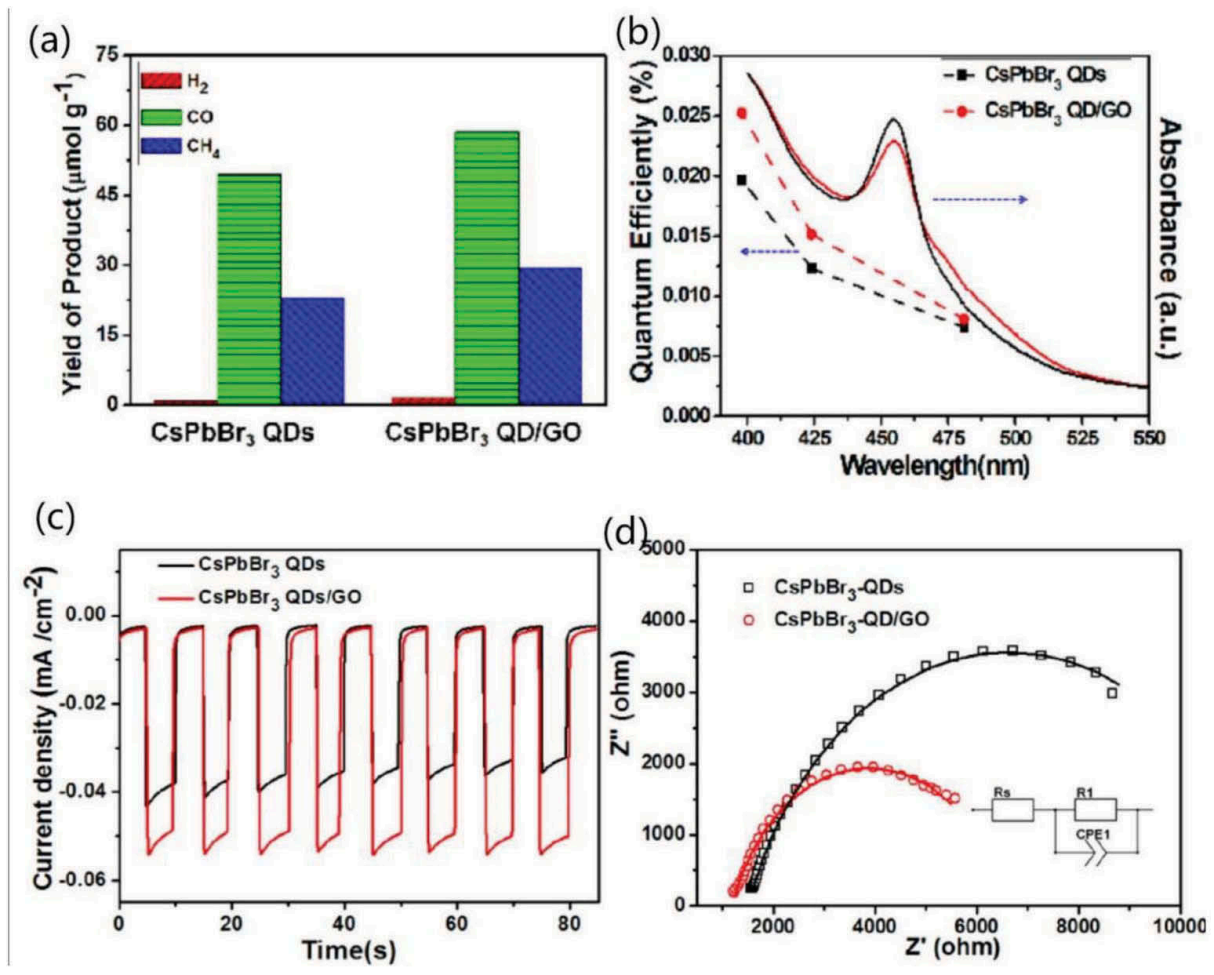
(a) Yield of product from CO_2_ reduction and (b) quantum efficiency using CsPbBr_3_ QD and CsPbBr_3_ QD/GO composite as the photocatalyst, respectively. (c) I-t curves under −0.4 V and electrochemical impedance Nyquist plots under 150 mW/cm^2^ and −0.4 V. Reproduced with permission from [116]. © 2017 ACS Publications.

For HP-QDs based composites, the structure of HP-QDs loaded on the surface of the matrix were preferred for the application in photocatalyst since the heterojunction structure of QDs with functional materials could not only improve the stability but also reach higher electron transportation efficiency. CsPbBr_3_ QD/GO composite was firstly studied in catalysing CO_2_ reduction into solar fuels via injecting electrons into CO_2_, reaching selectivity over 99.3% and improved charge consumption rate [116]. In that work, enhanced EQE and similar light absorbance of the composites compared to pure HP-QDs were observed (Figure 11a,b), indicating that the enhancement in efficiency mainly came from enhanced charge separation and transportation efficiency [167]. This was further confirmed by the tests on photoelectrochemical performances, where improved photocurrent response and reduced charge-transfer resistance were observed (Figure 11c,d). And, by using ethyl acetate as the solvent for CO_2_, where ethyl acetate could help stabilize the QDs, no degradation was observed after working 12 h [116]. Other composites as CsPbBr_3_/g-C_3_N_4_ nanosheet with chemical bonding [168], HP-QDs/metal-organic framework (MOF) with outstanding yield of 1559 μmol/g were also reported [169,170]. Another composite Ag-CsPbBr_3_/CN was fabricated and reported by Zhao et al. to degrade 7-aminocephalosporanic acid and exhibited outstanding catalytic activity due to the reduced charge recombination, improved charge-separation efficiency and light absorption of the whole composite structure [115].

**Figure 12.**
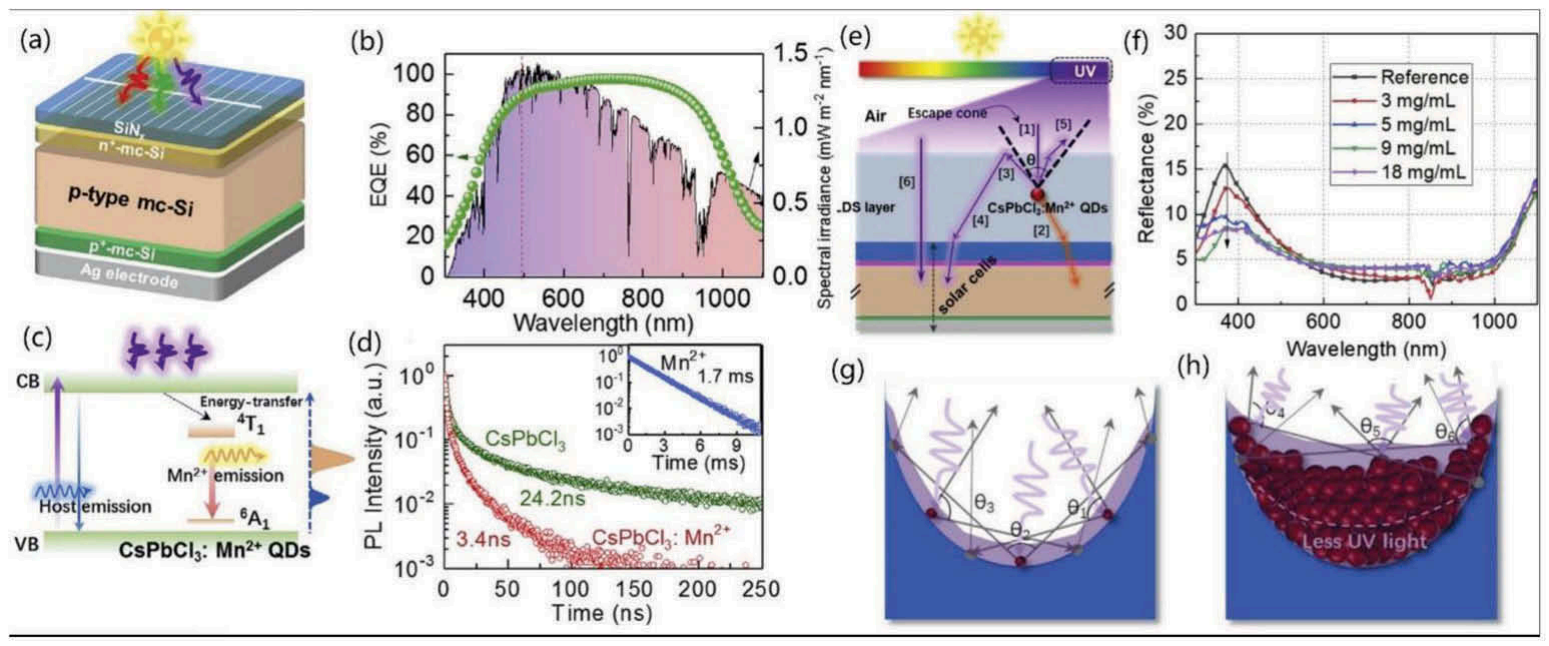
(a) Schematic illustration and (b) EQE of traditional silicon solar cell. (c) Energy level diagram of Mn^2+^:CsPbCl_3._ (d) Working lifetime of pure Mn^2+^, CsPbCl_3_ QDs and Mn^2+^:CsPbCl_3_ QDs. (e) Schematic illustration and (f) Surface reﬂectance spectra of silicon solar cell with Mn^2+^:CsPbCl_3_ layer. Influence of Mn^2+^:CsPbCl_3_ QDs layer on second-reflection with different concentration. (g) 3 mg/mL and (h) 18 mg/mL. Reproduced with permission from [122]. © 2018 ScienceDirect.

As for photovoltaic, devices with high power conversion efficiency (PCE), low cost and great stability are required. In perovskite solar cell, introducing composite materials could help improve the phase stability and carrier mobility of the HP-QDs. For example, Sanehira et al. adopted A-site cation halide salt (AX)-coated CsPbI_3_ QDs where AX salt treatment could double the mobility, enhance the photocurrent and achieve highest PCE at 13.4% and short-circuit current density (*J_SC_*) of 14.37 mA/cm^2^ [171]. And in silicon solar cell (Figure 12a), the HP-QDs based composites could be used as luminescent downconverter layer [172], such as Mn^2+^:CsPbCl_3_ [122,173]. The efficiency of the original silicon solar cell (Figure 12b) was low for short wavelength because of the undesirable parasitic optical absorption, which resulted in recombination loss and limited the device performance [174]. Using Mn^2+^:CsPbCl_3_ as the composite layer on the front of silicon, the electrons excited by UV light would then relax to the lower band of Mn^2+^ (^4^T_1_, as shown in Figure 12c). Thus, with a large Stokes shift (200 nm) and high PL quantum yield (62%), this composite layer could convert UV light into visible light (Figure 12d). With increasing concentration of Mn^2+^:CsPbCl_3_, the surface reflectance of the device on UV light significantly decreased, indicating that more UV light was absorbed and then converted (Figure 12e-h) to reach a higher PCE. This device obtained enhanced *J_SC_* (by 5.1%) and PCE (by 6.2%) compared to the original silicon device [122].

### 3.5. Memristor

**Figure 13.**
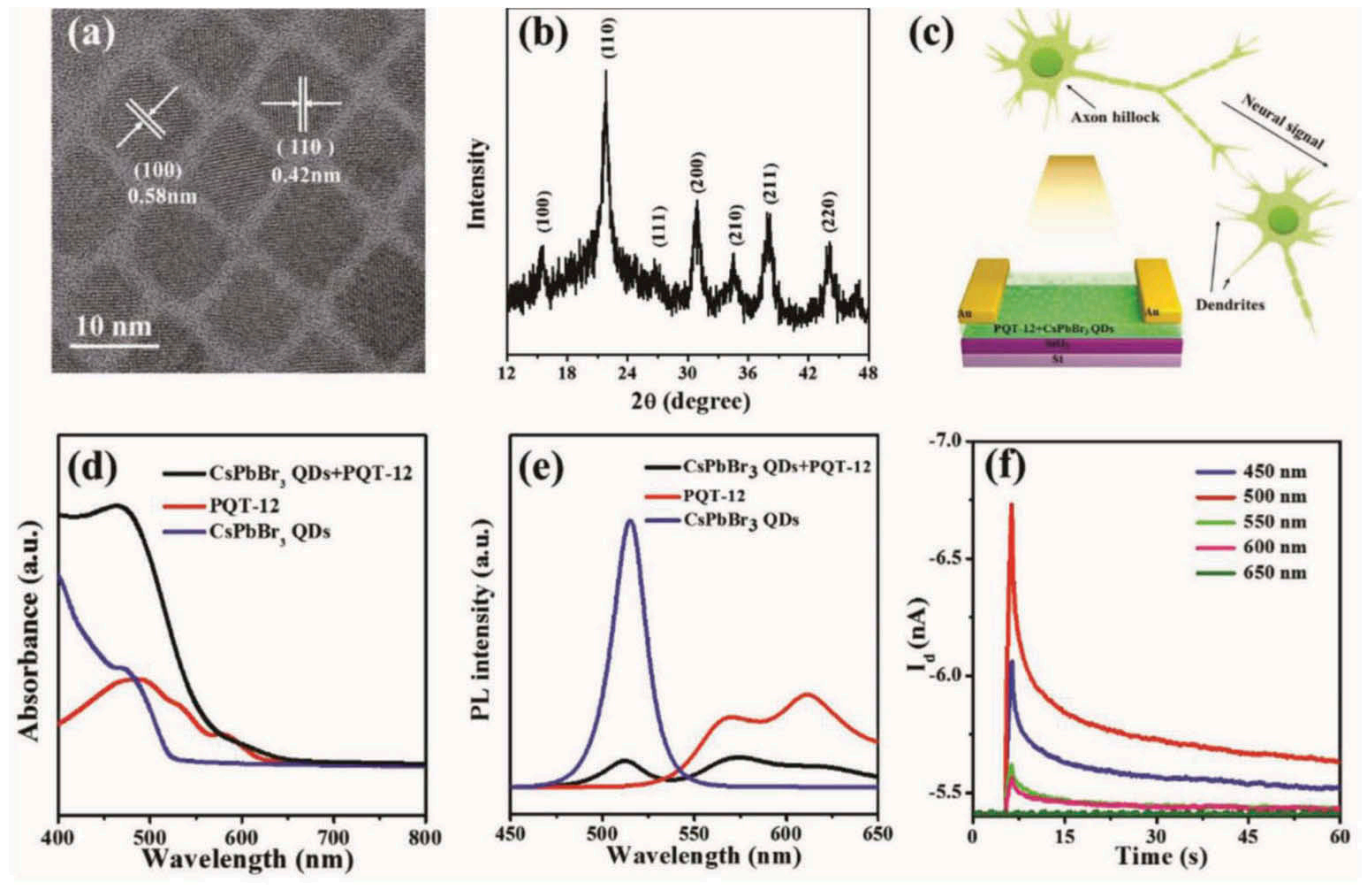
(a) TEM image and (b) XRD pattern of pure CsPbBr_3_ QDs. (c) Schematic illustration of the device structure. (d) Optical absorption and (e) PL spectrum of pure CsPbBr_3_ QDs, PQT-12 and CsPbBr_3_ QDs/PQT-12 composites. Optical response of CsPbBr_3_ QDs/PQT-12 composites. Reproduced with permission from [178]. © 2019 Wiley.

Possessing excellent photoelectronic properties, HP-QDs were also considered as promising candidates for memristor via light-stimulated resistive switching; however, instability to environmental factors, low electron-transport efficiency and easy interfacial reaction with electrode layer limit their application [175,176]. Stable devices with high photoresponsivity and efficiency are required for light-stimulated memristor. Forming the composite structure of HP-QDs encapsulated in matrix such as PMMA is an efficient strategy in improving the stability. Adopting CsPbCl_3_/PMMA composite as the active layer, the memristive device exhibited improved retention time (10^4^ s) [177]. HP-QDs/organic-semiconductor (poly(3,3-didodecylquarterthiophene) ‘PQT-12’ was used here) composite in this application was also studied, where HP-QDs/PQT-12 composite film was utilized as light-absorbing and charge-transporting layer (Figure 13a-c) [178]. Compared to pure HP-QDs and pure PQT-12, the composite exhibited enhanced optical absorption of a wider spectrum (Figure 13d), quenched PL intensity for reduced rate of carrier recombination (Figure 13e). With these merits and the disordered interfaces of the composite, this device exhibited improved charge-separation efficiency and induced delayed decay (Figure 13f) for higher photoresponsivity and efficiency [178].

Loading HP-QDs on the material’s surface is another method to protect the HP-QDs, where the protection layer such as PMMA could be formed on both sides of HP-QDs [179,180]. And for functional oxide materials such as ZnO, not only could the oxide layer prevent HP-QDs from contacting the electrode but also it help form HP-QDs/ZnO heterojunction to achieve enhanced rapid response speed (<1 ms) [181].

## 4. Summary

Here, we have presented various works on HP-QDs based composites with different structures and enhanced properties. On the one hand, to enhance the stability of pure HP-QDs, two strategies of compositing were explored. By encapsulating HP-QDs into material matrix or more precisely core-shell structure, the protection materials including oxides, glass, organic polymers, salts, semiconductors and MOFs could enhance the stability against environmental factors and maintain their unique optical properties at the same time. Template-free methods with outstanding monodispersity and template method having better control in size confinement of the QDs are all discussed. And by ion-doping, the lead in the perovskite lattice was replaced by mental ions to some extend to improve its formation energy and by so enhance the thermal stability. On the other hand, heterojunctions with various functional materials for enhanced optical or photoelectronic properties were obtained in composites via loading HP-QDs on the surface physically by spin-coating or chemically by linker molecules and HP-QDs/QDs structures.

These composites exhibiting enhanced stability and photoelectronic properties enabled HP-QDs to be better applied in various applications including photo-emission, photocatalytic, photodetector, photovoltaic, light-stimulated memristor and some chemical sensors. Benefit from the protecting layer with much lower penetration rate of molecules from the environment, the HP-QDs/matrix composites with great water-resistance, long-term stability and reduced anion-exchange effect shows promising potential in LED and WLED. Ion-doping for enhanced thermal stability and modulated PL kinetics have also attracted attentions in LED and solar cell for high PL efficiency, light conversion efficiency and reduced Pb toxicity of their lattice structure. By forming heterojunctions of HP-QDs with other functional materials, the composites could exhibit different merits for various chosen materials. Oxides with different energy band for HP-QDs/oxide heterostructures may induce limited electron injection for intense light emission in LED or enhanced electron injection for great performance in photocatalytic and photodetector, and semiconductor materials with enhanced efficiency in charge separation and transport can be used in light-stimulated memristor. However, protecting matrix layer that isolates the QDs well would unavoidably limit its photoelectronic performance to some extent, and functional materials forming heterojunctions with enhanced photoelectronic properties always have little contribution to the instability issue of HP-QDs. To combine the merits of different structures for a better performance in application, various matrix materials for better maintained properties, composites with more than one structure mentioned above, and functional materials that could both form heterojunction and play as capping matrix have all been explored. Recent examples include 1) paraffin as encapsulating material with high transparency to UV light that can maintain the high PL quantum yield of HP-QDs [182], 2) poly(ethylene oxide) as matrix to both protect the ligands and deactivate the defects at the QDs surface [183] and 3) MOF materials that enhance the stability and carrier-transport efficiency of HP-QDs [169,170]. We believe that composites with better performance will be achieved in the future.
